# Magnetic resonance spectroscopy for quantification of liver iron deposition in hereditary hemochromatosis of a Chinese family: Four case reports

**DOI:** 10.1097/MD.0000000000031742

**Published:** 2022-11-18

**Authors:** Jing Zhang, Kefu Liu, Yan Sun, Jiafeng Yu

**Affiliations:** a Department of Radiology, The Affiliated Suzhou Hospital of Nanjing Medical University, Suzhou, China.

**Keywords:** hereditary hemochromatosis, HISTO sequence, iron deposition, magnetic resonance imaging

## Abstract

Hereditary hemochromatosis (HH) is a major cause of liver iron overload. The gold standard for the diagnosis of liver iron overload is the histopathological analysis of a liver sample collected by biopsy. The biopsy procedure is both invasive and painful and carries some risks of complications. The multi-echo single-voxel magnetic resonance spectroscopy (HISTO) technique can be used for noninvasive, quantitative assessment of liver iron overload.

**Patient concerns:**

We report 4 Chinese Han men, who were relatives. Patient A was admitted with diabetes and presented with thrombocytopenia and skin hyperpigmentation. The other patients had no specific clinical presentation.

**Diagnoses:**

Patient A was suspected of having iron in the liver on routine magnetic resonance imaging, therefore, further HISTO, laboratory testing, and liver biopsy were performed, which confirmed iron metabolic abnormalities. Furthermore, we identified hepatic iron deposition using HISTO and laboratory testing of his son and 2 brothers. Combined with symptoms, auxiliary examinations, and liver biopsy, HH was considered.

**Interventions:**

As the 4 patients had no other discomfort other than patient A who had diabetes, patient A was placed on therapy comprising the insulin pump, acarbose, and platelet booster capsule.

**Outcomes:**

After treatment, the diabetic symptoms of patient A improved. The patient and his relatives were regularly followed-up for HH.

**Lessons:**

HH should be considered when hepatic iron deposition is suspected by routine magnetic resonance, as the HISTO sequence can quantitate liver iron deposition and leads to a promising diagnosis. HISTO is of great value in familial cases, especially in young patients requiring long-term follow-up.

## 1. Introduction

Hereditary hemochromatosis (HH) is an iron-storage disease, caused by mutations in genes involved in the regulation of iron homeostasis.^[1–3]^ Liver iron concentration (LIC; mg Fe/g dry liver tissue) is a surrogate for total body iron stores.^[4]^ In untreated individuals, iron overload can lead to liver fibrosis and cirrhosis. The early recognition, diagnosis, and treatment of HH can reduce iron deposition and prevent disease progression. Due to its invasive nature, high inter-/intra-observer variability, and sampling bias, liver biopsy is no longer required for HH diagnosis.^[5,6]^ Magnetic resonance imaging (MRI) has been used as a standard imaging modality for the detection, quantification, and monitoring of iron overloads.^[7,8]^ However, there are different MR techniques for iron quantification and there is still no consensus regarding the best technique or postprocessing tool for hepatic iron quantification.^[4]^

*R*2 and *R*2* relaxometries are widely used approaches for liver iron quantification.^[4,9]^ Ferriscan-*R*2 is the conventional technique, based on spin-echo measurement, used for hepatic iron quantification. However, long acquisition times (approximately 20 minutes), complex data analyze, and increased costs limit its use.^[4]^ The latest *T*2-corrected multi-echo single-voxel magnetic resonance spectroscopy (HISTO) sequence uses one-breath-holding technology and allows safe, noninvasive diagnosis and monitoring of liver iron deposits.^[10]^

Here we report four patients with HH from a Chinese Han family who were quantified for liver iron overload using the HISTO sequence.

## 2. Case report

### 2.1. Patient A

A 55-years-old man presented with elevated blood glucose levels for 1 year and poor glucose control for 1 week. Five years previously, the patient had a history of thrombocytopenia. The patient had been admitted to another hospital for fasting blood glucose levels up to 18 mmol/L 1 year before presentation at our hospital, where he was diagnosed with diabetes, splenomegaly, and thrombocytopenia, and his blood glucose was poorly controlled after treatment with insulin aspart 30 and acarbose. The patient denied tobacco or alcohol use, and had no history of surgery, blood transfusion, or hemolysis. History of hypertension, pancreatitis, hepatitis, and schistosomiasis were not found. Physical examination revealed skin hyperpigmentation.

**Figure 1.**
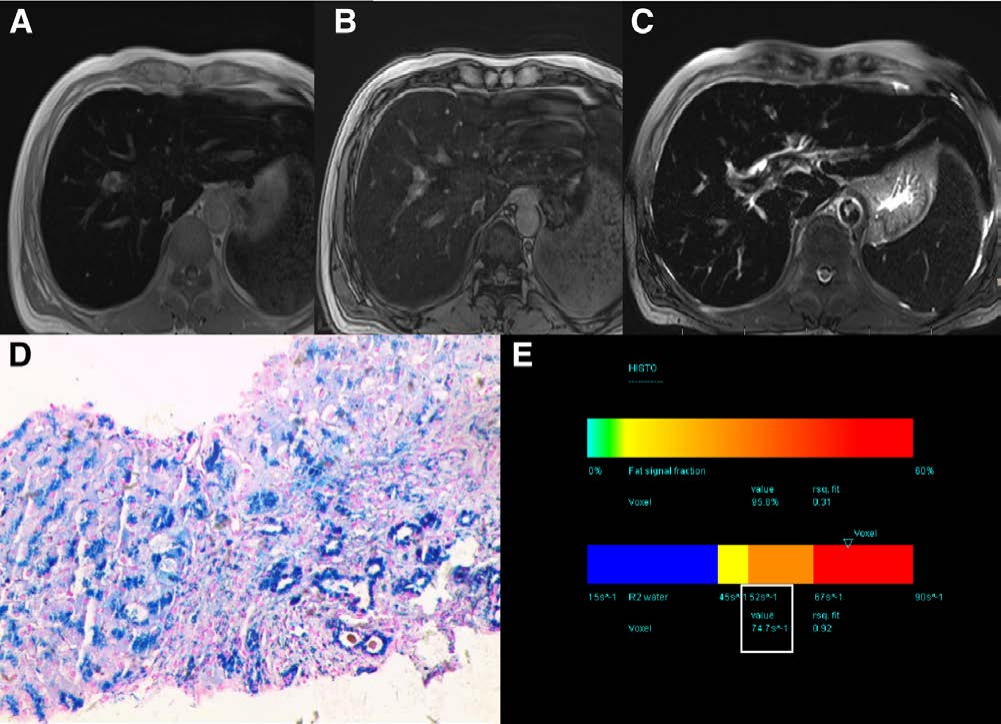
Techniques for detecting liver iron deposits in Patient A. (A and B) T1 VIBE two-point Dixon technique showed lower signal intensity on in-phase than opposed-phase images. (C) T2 TSE fs technique revealed a significant low signal. (D) HE showed pigmentary particles deposition in the hepatocyte cytoplasm with refractivity (HE × 200). (E) MR spectroscopy at multiple echo times with T2 curve fit of water and lipid spectrum calculates the R2_water_ (white box). HE = hematoxylin and eosin staining, MR = magnetic resonance, TSE = turbo spin echo, VIBE = volumetric interpolated breath-hold examination.

The routine MRI scan showed low signal intensity on *T*2 weighted images and lower in-phase than out-of-phase signal intensity in the whole liver. Iron was suspected to have been deposited in the liver; therefore, a further HISTO method was performed (Fig. 1). The results revealed that the value of R2_water_ was 74.7 s^-1^ (reference range, 15–90 s^-1^). Based on the radiological findings, the patient underwent other tests, including blood testing and liver biopsy. Liver function tests showed liver dysfunction (Table 1). A very high serum ferritin concentration (>1500 ng/mL) (reference range, 23.9–336.2 ng/mL) was found in iron metabolism. Iron metabolism also showed a transferrin of 1.30 g/L (reference range, 2.0–3.6 g/L), total iron binding capacity of 32.10 µmol/L (reference range, 50.0–77.0 µmol/L), and transferrin saturation of 95.0% (reference range, 20–55%). Liver biopsy showed chronic inflammatory cell infiltration in the confluent area and diffuse iron-containing hematoxylin deposits in the central lobule and confluent area.

**Table 1:** Liver function data of the patient A.

	Measured data	Reference level
ALT	77 U/L	9–50 U/L
AST	62.2 U/L	15–40 U/L
AKP	194 U/L	45–125 U/L
*γ*-GT	93.7 U/L	10–60 U/L
TBIL	26.2µmol/L	0–26µmol/L
ALB	42.9 g/L	40–55 g/L

*γ*-GT = *γ*-glutamyl transpeptidase, AKP = alkline phosphatase, ALB = albumin, ALT = alanine aminotransferase, AST = aspartate aminotransferase, TBIL = total bilirubin.

The patient was treated with an insulin pump combined with acarbose (50 mg, bid) for his secondary diabetes and a platelet-booster capsule for thrombocytopenia. For HH, the patient agreed to regular follow-up as there were no associated symptoms.

Regarding the patient’s family history, his father died when he was 4 years old, and his mother died at the age of 85. He had 1 older sister and 3 older brothers. His sister died of unknown causes and his oldest brother died of esophageal cancer. The other 2 older brothers were all alive. In addition, he had 1 son. His son and 2 older brothers were then sent for blood testing and MRI scans, and the values are shown in Table 2.

**Table 2:** Serum transferrin and iron metabolism data for patients B, C, and D.

	Patient B	Patient C	Patient D	Reference level
Serum iron	18.6 µmol/L	41 µmol/L	17.2 µmol/L	10.6–36.7 µmol/L
Total iron binding capacity	49.7 µmol/L	41 µmol/L	49.5 µmol/L	50–77 µmol/L
Transferrin saturation	37%	100%	35%	20–55%
Serum ferritin concentration	218.2 ng/mL	>1500 ng/mL	270.8 ng/mL	23.9–336.2 ng/mL

### 2.2. Patient B

**Figure 2.**
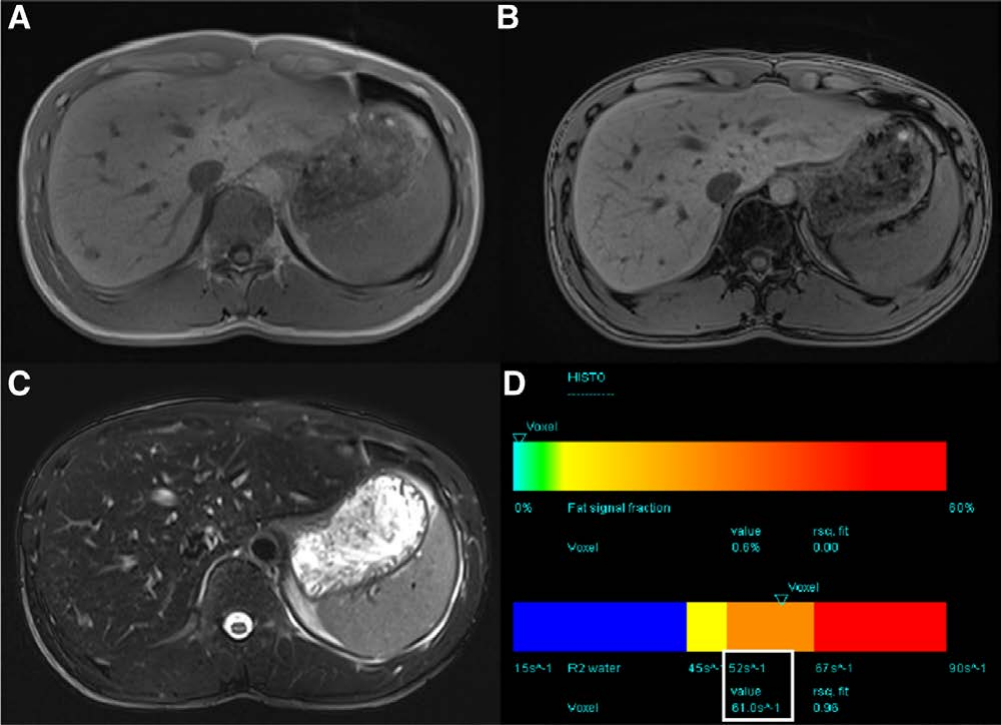
Techniques for detecting liver iron deposits in Patient B. (A and B) T1 VIBE two-point Dixon technique showed slightly lower signal intensity on in-phase than opposed-phase images. (C) T2 TSE fs technique revealed a slightly low signal. (D) MR spectroscopy at multiple echo times with T2 curve fit of water and lipid spectrum calculates the R2_water_ (white box). MR = magnetic resonance.

A 33-years-old man was the son of a previous patient. Iron metabolism showed that his total iron-binding capacity was mildly reduced. Serum iron transferrin saturation and serum ferritin levels were normal. The liver showed a slightly lower signal intensity on *T*2-weighted images (Fig. 2). In addition, the signal intensity of the liver on in-phase (second echo) images was also slightly lower than that on opposed-phase (first echo) images. In the further HISTO test, R2_water_ was 61.0 s^-1^. The patient showed no other clinical symptoms.

### 2.3. Patient C

**Figure 3.**
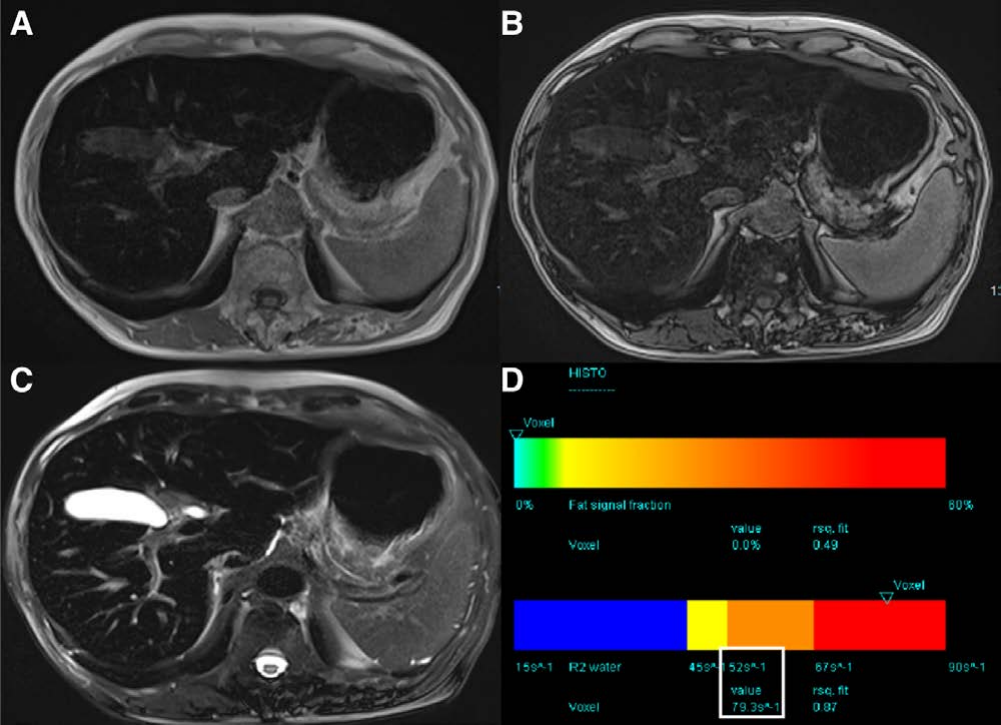
Techniques for detecting liver iron deposits in Patient C. (A and B) T1 VIBE two-point Dixon technique showed lower signal intensity on in-phase than opposed-phase images. (C) T2 TSE fs technique revealed a significant low signal. (D) MR spectroscopy at multiple echo times with T2 curve fit of water and lipid spectrum calculates the R2_water_ (white box). MR = magnetic resonance.

A 67-years-old man, the third brother of Patient A. Blood testing revealed an extremely high serum ferritin concentration, high serum iron, and transferrin saturation. Total iron-binding capacity was below the normal range. His liver behaved similarly to patient A in routine MR (Fig. 3). The R2_water_ was 79.3 s^-1^.

### 2.4. Patient D

**Figure 4.**
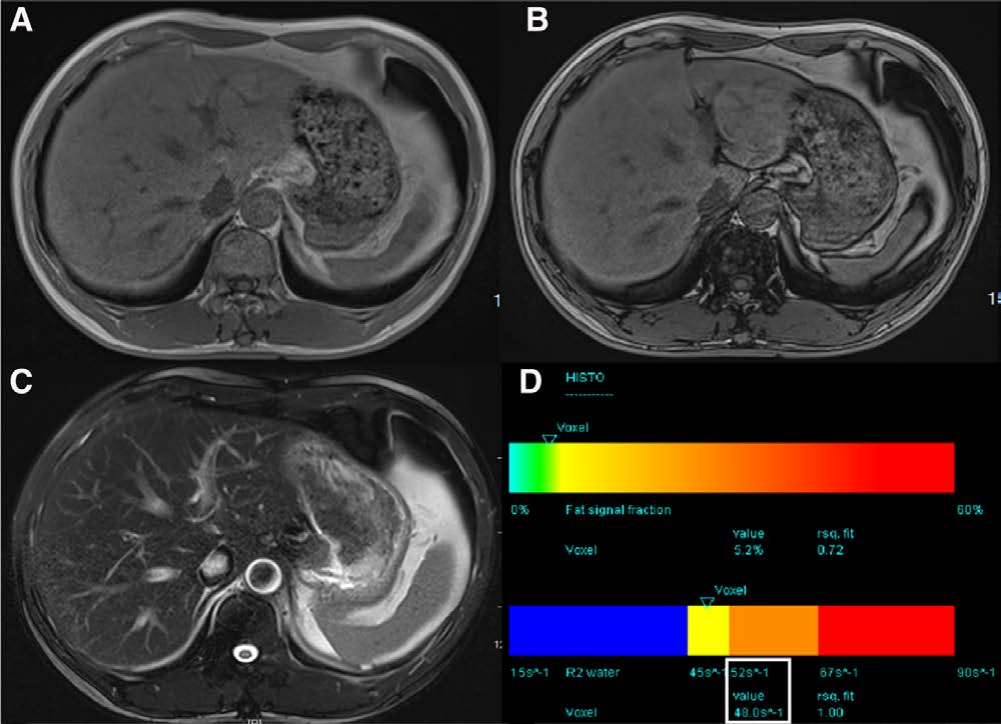
Techniques for detecting liver iron deposits in Patient D. (A and B) T1 VIBE two-point Dixon technique showed slightly lower signal intensity on in-phase than opposed-phase images. (C) T2 TSE fs technique revealed a slightly low signal. (D) MR spectroscopy at multiple echo times with T2 curve fit of water and lipid spectrum calculates the R2_water_ (white box). MR = magnetic resonance.

A 71-years-old man, the middle brother of patient A. His blood tests and conventional MR were similar to those of patient B, and only the total iron-binding capacity was slightly lower than the normal values. In the HISTO sequence, R2_water_ was 48 s^-1^(Fig. 4).

## 3. Discussion

In this report, Patient A was admitted with diabetes and had an abnormal liver signal on routine MRI. On the basis of the suspicion of iron deposits in the liver, a further HISTO scan was performed. The R2_water_ value was confirmed by heavy iron deposition. Laboratory tests also showed significantly elevated serum ferritin and transferrin saturation. HH was basically considered. On this basis, laboratory tests and MR examinations were performed on his son and 2 brothers, and iron deposits were found in their livers. The HH hypothesis was confirmed.

HH is an iron-storage disease, caused by mutations in the genes involved in the regulation of iron homeostasis.^[11-–13]^ The liver is the main organ responsible for iron storage.^[14]^ Quantitative measurement of liver iron concentration helps detect systemic iron overload and monitor the effectiveness of clinical treatment.^[4,15]^ Therefore, liver biopsy is the reference standard for quantifying hepatic iron and assessing body iron stores.^[16]^ However, owing to its invasive nature, high inter-/intra-observer variability, and sampling bias, MR has become a noninvasive method for qualitative and quantitative assessment of liver iron deposition.^[17]^ Studies have shown that MR techniques for detecting iron deposits in the liver correlate well with liver biopsies.^[10,18,19,20]^

The presence of iron results in a liver signal loss on *T*2 and *T*2* weighted images, which shows low signal intensity on *T*2 and *T*2* weighted images. In addition, the lower signal intensity in the in-phase than in the opposed-phase can help identify liver iron deposits.^[4,9]^ However, these sequences cannot quantify iron deposition, and concomitant steatosis reduces the value of the use of in- and opposed-phases.

In recent years, several MR sequences have been developed for quantitative liver iron deposition, with *R*2 and *R*2* relaxometry being the most frequently used techniques in clinical practice.^[21,22]^ The *R*2* relaxometry, which is based on gradient-echo, is a fast acquisition, with the possibility of 3D acquisitions and parallel imaging (covering the whole liver in a single acquisition).^[20,23,24]^ In addition, the results were highly reproducible. However, *R*2* measurements may be affected by liver fibrosis or coexisting fat.^[25]^ Recently, a multi-echo Dixon sequence was developed to overcome these difficulties. Some studies have demonstrated a good correlation between the 3D multi-echo Dixon and 2D gradient-echo techniques and biopsy.^[10,26]^ The multi-echo Dixon method allows the placement of regions of interest or automatic whole-liver analysis.^[22]^ Some inaccuracies were observed for multi-echo Dixon imaging in cases of very high LIC, mainly at 3T.^[18]^

HISTO is a T2-corrected multi-echo single-voxel MR spectroscopy sequence that acquires multiple echoes in a single acquisition within one breath-hold.^[10,19,27]^ We use this sequence to quantify liver iron deposition because of its inherent ability to separate water resonance from other confounding sources of proton resonance. Zhan et al reported that the ability of MRS to detect hepatic iron overload is equal to that of multi-echo Dixon at 3T.^[10]^ In addition, a study also showed that the modified MRS sequence at 1.5T had a strong correlation between MRS-R2_water and FerriScan-R2, which is a regulatory-approved standardized spin-echo measurement.^[27]^ In our study, high-speed T2-corrected multi-echo single-voxel spectroscopy (HISTO) was used to evaluate the LIC in HH, and the value of R2_ water_ was in agreement with the liver biopsy and iron metabolism.

The cases described in this report provided several conclusions. First, HISTO is of great value in quantifying liver iron deposition, consistent with elevated transferrin saturation, serum ferritin concentrations, and liver biopsy. Second, in the clinical setting, routine MRI was aimed at incidental findings in the general patient population. If liver iron deposition is suspected, quantitative techniques, such as HISTO, will always be performed. Third, for patients with confirmed HH, asymptomatic members of their families may use HISTO to assist in diagnosis. In familial cases, especially in young patients who require long-term follow-up, HISTO can be used to avoid invasive tests. Finally, this technique can be used to evaluate the effectiveness of the treatment.

## Author contributions

**Conceptualization:** Kefu Liu.

**Data curation:** Jing Zhang

**Formal analysis:** Jing Zhang.

**Funding acquisition:** Kefu Liu.

**Investigation:** Jing Zhang.

**Methodology:** Jing Zhang.

**Resources:** Jing Zhang, Jiafeng Yu.

**Software:** Yan Sun.

**Supervision:** Jing Zhang.

**Validation:** Jing Zhang.

**Visualization:** Jing Zhang.

**Writing – original draft:** Jing Zhang.

**Writing – review & editing:** Kefu Liu.
